# Risk Prediction and Management of BKPyV-DNAemia in Kidney Transplant Recipients: A Multicenter Analysis of Immunosuppressive Strategies

**DOI:** 10.3389/ti.2025.14738

**Published:** 2025-07-17

**Authors:** Jin-Myung Kim, Hye Eun Kwon, Ahram Han, Youngmin Ko, Sung Shin, Young Hoon Kim, Kyo Won Lee, Jae Berm Park, Hyunwook Kwon, Sangil Min

**Affiliations:** ^1^Division of Kidney and Pancreas Transplantation, Department of Surgery, Asan Medical Center, University of Ulsan College of Medicine, Seoul, Republic of Korea; ^2^Division of Transplantation and Vascular Surgery, Department of Surgery, Seoul National University Hospital, Seoul, Republic of Korea; ^3^Department of Surgery, Samsung Medical Center, Sungkyunkwan University School of Medicine, Seoul, Republic of Korea

**Keywords:** kidney transplantation, Bk virus, immunosuppressive therapy, calcineurin inhibitor, tacrolimus trough level

## Abstract

BK polyomavirus (BKPyV) DNAemia remains a major complication in kidney transplantation (KT), requiring nuanced adjustments to immunosuppressive regimens to control viral replication while minimizing rejection risk. This retrospective multicenter cohort study included 8,027 KT recipients, of whom 1,102 developed BKPyV-DNAemia within the first year. Among them, 927 patients with complete therapeutic drug monitoring (TDM) data were categorized into three groups based on post- BKPyV-DNAemia immunosuppressive strategies: mycophenolic acid (MPA) control, sirolimus, and leflunomide. Multivariate logistic regression and Cox analyses identified risk factors for BKPyV-DNAemia treatment failure, acute rejection, and graft loss. Tacrolimus trough levels below 5 ng/mL and complete withdrawal of calcineurin inhibitors (CNIs) significantly increased rejection risk (OR = 2.65, P = 0.033). Maintaining tacrolimus levels between 5 and 7 ng/mL was associated with optimal viral control and lower rejection rates. Leflunomide substitution reduced BKPyV burden but increased rejection risk (OR = 2.14, P < 0.001). Sirolimus-based regimens with CNI withdrawal led to the highest rejection risk (OR = 6.00, P = 0.044) and a trend toward increased graft failure (HR = 4.37, P = 0.07). A tacrolimus target of ≥5 ng/mL emerged as optimal for balancing BKPyV-DNAemia suppression and long-term graft survival. While leflunomide is effective for viral control, its immunological risks warrant careful patient selection and monitoring.

## Graphical Abstract

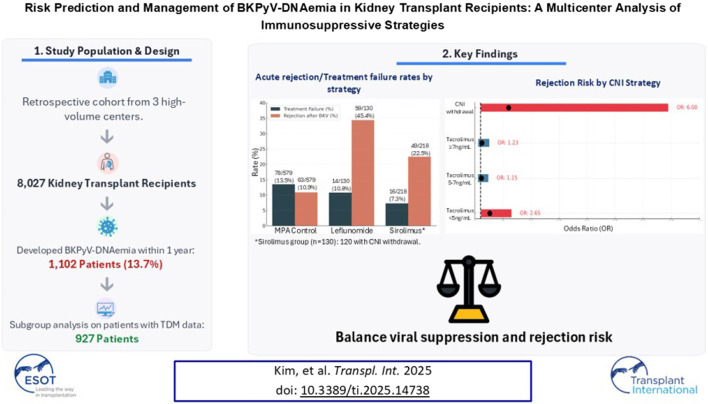

## Introduction

Kidney transplantation (KT) is a vital treatment option for patients with end-stage renal disease, significantly improving both survival rates and quality of life [1, 2]. Despite its many advantages, post-transplant complications continue to pose challenges to graft longevity and patient outcomes [3, 4]. Among these complications, BK polyomavirus (BKPyV) DNAemia is recognized as a major concern affecting post-transplant outcomes [5–7]. The BKPyV, a member of the polyomavirus family, typically remains latent in renal tissue [8]. However, under conditions of immunosuppression, which are necessary to prevent graft rejection, the virus can reactivate [9]. This reactivation may lead to BK virus-associated nephropathy (BKVN), which is a leading cause of graft dysfunction.

The management of immunosuppression in KT recipients presents a critical clinical dilemma. Immunosuppressants, particularly calcineurin inhibitors (CNI) such as tacrolimus and mycophenolic acid (MPA), are essential for preventing organ rejection [10]. However, these same medications may inadvertently promote viral reactivation [11]. The challenge lies in reducing immunosuppression to mitigate the risk of BKPyV-DNAemia while simultaneously maintaining adequate immunosuppression to prevent rejection. Previous research has underscored the importance of maintaining optimal tacrolimus levels to maximize graft survival [12]. The present study builds upon this foundational work by offering a detailed analysis of risk factors, refining tacrolimus thresholds, and evaluating the efficacy of alternative immunosuppressive strategies that can minimize complications related to the BKPyV.

As the number of immunologically high-risk KT recipients continues to rise, BKPyV-DNAemia has become an increasingly critical concern for graft survival [13]. However, large-scale, multicenter studies addressing this issue are limited, and there is a notable lack of research on the relationship between CNI concentration and BKPyV-DNAemia outcomes. By leveraging clinical data from a large multicenter cohort, our study aims to establish the most effective immunosuppressive management following BKPyV-DNAemia onset by defining appropriate CNI trough levels and assessing the impact of different immunosuppressive regimens—such as leflunomide and sirolimus—on viral control, rejection risk, and long-term graft survival. Additionally, we seek to identify significant predictors and risk factors for BKPyV-DNAemia, enabling early detection and targeted intervention.

## Materials and Methods

### Study Design and Population

**FIGURE 1:**
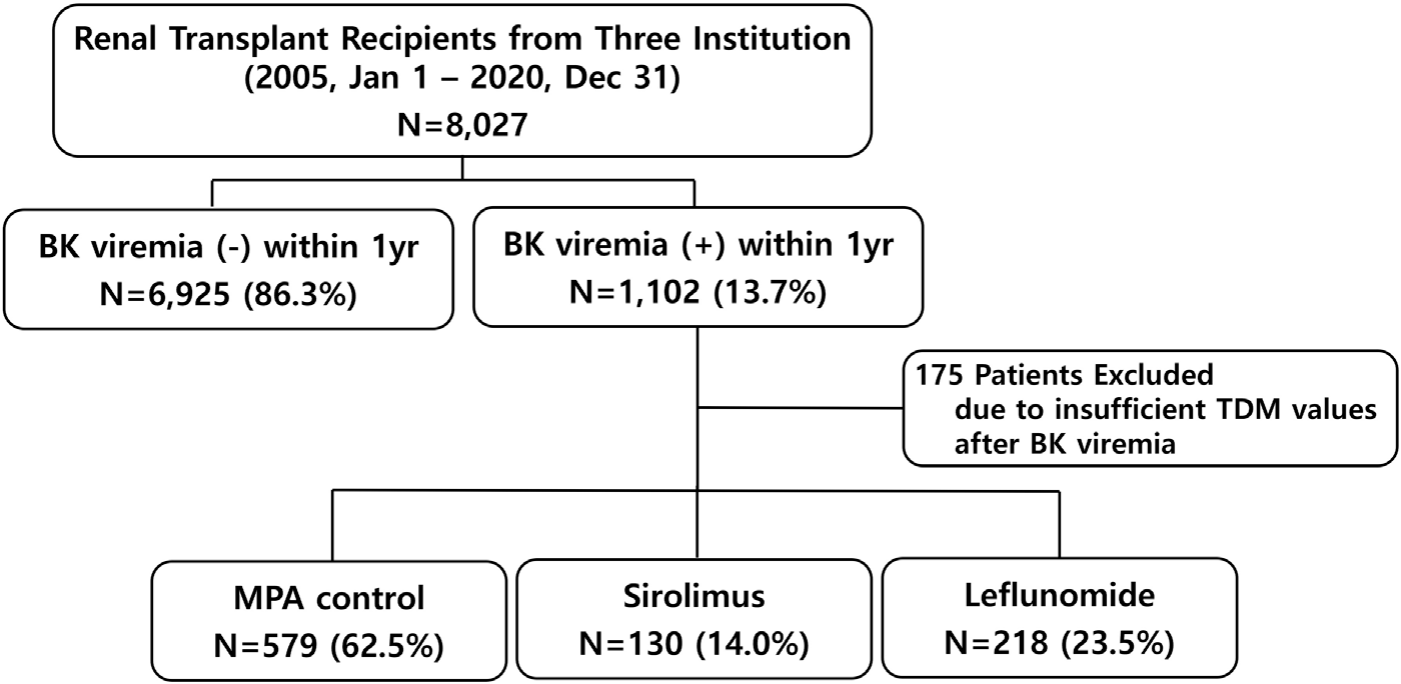
Patient selection flowchart.

This retrospective cohort study analyzed data collected over 15 years (2005–2020) from five transplant centers in South Korea that participated in a preceding study [12]. Of these five centers, only three had complete raw data on BKPyV; therefore, the final study population was limited to these three high-volume transplant centers. To ensure data integrity and relevance, strict inclusion and exclusion criteria were applied. Adult KT recipients (≥18 years of age) with at least 1 year of post-transplant follow-up were eligible for inclusion. A total of 8,027 recipients from the three institutions were included based on the inclusion criteria. For the subgroup analysis, 927 patients were selected after excluding those with missing therapeutic drug monitoring (TDM) data for CNI following the onset of BKPyV-DNAemia. These patients were then categorized into three groups based on their post-viremia immunosuppressive management strategies (Figure 1). The study was conducted in accordance with the principles outlined in the Declaration of Helsinki and was approved by the Institutional Review Board of Asan Medical Center (IRB number: 2022-0139).

### Data Collection and Processing

Data were extracted from centralized electronic medical records at the three participating centers using institutional clinical data warehouses. To ensure consistency, the investigators collaboratively defined key variables and operational definitions. Custom extraction algorithms facilitated the automated collection of recipient and donor demographics, transplant details, laboratory results, medication histories, and clinical outcomes. For this study, additional analyses were conducted using a refined dataset from a previous study [12], focusing specifically on raw data related to BKPyV-DNAemia, tacrolimus TDM results, and immunosuppressant prescription histories. All participating centers used quantitative polymerase chain reaction (qPCR) assays to monitor BKPyV-DNAemia in plasma specimens collected in EDTA tubes. While minor changes in assay platforms or reagents occurred over the 15-year study period due to technological updates, each center maintained internal quality control and calibration procedures to ensure consistency in viral load reporting. Inter-laboratory variability was minimized by interpreting BKPyV-DNAemia trends relative to each patient’s baseline within the same institution, rather than applying absolute viral load cutoffs across centers.

### Immunosuppressive Regimen and BKPyV-DNAemia Monitoring

The three participating institutions utilized similar immunosuppression protocols for KT, including maintenance immunosuppression and infection prophylaxis, with detailed methodologies referenced in prior studies [12, 14, 15]. For pretransplant desensitization in ABO- and HLA-incompatible recipients, rituximab (100–500 mg; Genentech, Inc., South San Francisco, CA, USA) was administered 1–2 weeks prior to plasmapheresis (PP; COBE^®^ Spectra, Gambro BCT, Lakewood, CO, USA). PP continued until either IgM titers were ≤1:4 or IgG titers were ≤1:8 (ABOi), or until negative complement-dependent cytotoxicity crossmatch and T-cell flow-cytometric crossmatch (HLAi) were achieved. For induction therapy, basiliximab (20 mg on days 0 and 4) or anti-thymocyte globulin (ATG, 1.5 mg/kg/day) was used, with ATG reserved for high-risk patients. Maintenance immunosuppression consisted of a calcineurin inhibitor (tacrolimus or cyclosporin), mycophenolate mofetil (MMF), and corticosteroids. The detailed patterns and utilization of immunosuppressive agents among the study patients are described in a previous study [12]. At 2 months post-transplant, the most frequently observed tacrolimus trough level was ≥8.0 ng/mL in 40.0% of patients, followed by 7.0–7.9 ng/mL in 20.4% and 6.0–6.9 ng/mL in 16.6%. Notably, more than 60% of patients maintained an average tacrolimus trough level of at least 6.0 ng/mL for up to 10 months post-transplant.

At institutions participated in the present study, BKPyV monitoring was recommended at 1 and 2 weeks post-transplant, monthly until 6 months, and then every 2–3 months until 1 year post-transplant. However, testing intervals were adjusted in practice based on individual patient follow-up schedules and clinical judgment. Increased testing frequency was applied in cases of rising or high viral loads, while lower-risk patients were occasionally monitored less frequently. As such, the actual number of BKPyV-DNA tests per patient varied, and the total number of test results was substantially lower than the theoretical maximum. Across the three participating centers, a total of 34,355 BKPyV-DNAemia test results were obtained within the first post-transplant year for the 8,027 patients in this study (Center 1: 5,631 tests; Center 2: 4,562 tests; Center 3: 24,162 tests). This represents a substantial dataset for real-world BKPyV surveillance and supports the robustness of our virologic trend analysis.

### Definitions

HLA-incompatible KT was defined as transplantation in recipients with a positive complement-dependent cytotoxicity crossmatch and/or flow cytometric crossmatch. BKPyV-DNAemia positivity was identified as a log BKPyV PCR value greater than 3 within 1-year post-transplantation. Treatment failure was defined as a final follow-up log BKPyV PCR value greater than 3 persisting for at least 1 year after therapeutic intervention [16].

Subgroups were classified based on adjustments to primary immunosuppression following BKPyV-DNAemia detection. The Sirolimus group consisted of patients who transitioned from MPA to sirolimus within 6 months of BKPyV-DNAemia detection and remained on sirolimus-based therapy, including CNI withdrawal, for at least 6 months. Similarly, the Leflunomide group included patients who switched from MPA to leflunomide within 6 months of BKPyV-DNAemia detection and maintained leflunomide-based therapy for a minimum of 6 months. Lastly, the MPA control group included patients who underwent MPA tapering or discontinuation without transitioning to alternative therapies.

### Statistical Analysis

Risk factor analyses for BKPyV-DNAemia and treatment failure were performed using univariate and multivariate logistic regression models. These models were employed to estimate odds ratios (OR) and 95% confidence intervals (CI) to identify independent predictors. Variables with a P-value <0.1 in the univariate analysis were included in the multivariate models to adjust for confounding factors. Long-term clinical outcomes, including biopsy-proven acute rejection (BPAR)-free survival and the efficacy of different CNI management strategies, were assessed using Kaplan-Meier survival analysis, with log-rank tests employed for group comparisons. Cox proportional hazards regression was used to quantify hazard ratios (HR) and 95% CIs for risk factors affecting BPAR-free survival. For subgroup analyses, the associations between immunosuppressive regimens and clinical outcomes were examined using chi-square tests or Fisher’s exact tests for categorical variables, and Student’s t-tests or one-way analysis of variance (ANOVA) for continuous variables, as appropriate. Multicollinearity was evaluated using variance inflation factors, and covariate interactions were analyzed to improve interpretability. Statistical significance was set at P < 0.05, with results reported as ORs, HRs, or mean differences. Analyses were performed using IBM SPSS (version 22.0, IBM Corp., Armonk, NY, United States).

## Results

### Baseline Characteristics According to the Development of BKPyV-DNAemia

A total of 8,027 KT recipients from three centers met the inclusion criteria. Among these, 1,102 patients (13.7%) developed BKPyV-DNAemia within 1-year post-transplant. Table 1 compares the baseline characteristics of patients who developed BKPyV-DNAemia with those who did not. The BKPyV-DNAemia group was older (49.1 ± 12.9 years vs. 45.6 ± 14.2 years, *P* < 0.001) and had a higher body weight (61.7 ± 12.6 kg vs. 60.6 ± 14.0 kg, *P* = 0.016). The proportion of females was lower in the BKPyV-DNAemia group (38.1% vs. 41.7%, *P* = 0.024). Hypertension was more common in this group (81.8% vs. 77.8%, *P* = 0.003). Other notable characteristics of the BKPyV-DNAemia group include a higher prevalence of pre-transplant dialysis (*P* = 0.009), longer pre-dialysis duration (*P* < 0.001), and higher proportions of patients with ABO incompatibility (17.6% vs. 14.4%, *P* = 0.005), HLA incompatibility (7.2% vs. 5.4%, *P* = 0.018), and the use of ATG for induction therapy (25.3% vs. 18.3%, *P* < 0.001). Significant differences in CNI utilization were also noted (*P* = 0.005), with tacrolimus use being more prevalent in the BKPyV-DNAemia group.

**TABLE 1:** Baseline and clinical characteristics between kidney transplant recipients with and without BK viremia.

	BKPyV-DNAemia (−)	BKPyV-DNAemia (+)	P-value
Number of patients, n (%)	6,925 (86.3)	1,102 (13.7)	
Age, years (mean ± SD)	45.6 ± 14.2	49.1 ± 12.9	<0.001
Body weight, kg (mean ± SD)	60.6 ± 14.0	61.7 ± 12.6	0.016
Female, n (%)	2,888 (41.7)	420 (38.1)	0.024
Diabetes mellitus, n (%)	1777 (25.7)	307 (27.9)	0.12
Hypertension, n (%)	5,385 (77.8)	901 (81.8)	0.003
Pre-transplant dialysis, n (%)	5,738 (82.9)	948 (86.0)	0.009
Pre-dialysis duration, months (mean ± SD)	37.7 ± 49.7	44.3 ± 56.7	<0.001
ABO incompatibility, n (%)	997 (14.4)	194 (17.6)	0.005
HLA incompatibility, n (%)	374 (5.4)	79 (7.2)	0.018
Induction, n (%)			<0.01
none	375 (5.4)	13 (1.2)	
Basiliximab	5,251 (75.8)	806 (73.1)	
ATG	1,264 (18.3)	279 (25.3)	
Other^a^	35 (0.5)	4 (0.4)	
Calcineurin inhibitor, n (%)			0.005
Cyclosporin	2,511 (36.3)	366 (33.2)	
Tacrolimus	4,414 (63.7)	736 (66.8)	
Desensitization, n (%)	1,444 (20.9)	287 (26.0)	<0.001
Rituximab, n (%)	1,391 (20.1)	284 (25.8)	<0.001

Continuous data are presented as means ± standard deviations. Categorical data are presented as a number (%).

Abbreviations: ATG, anti-thymocyte globulin; TDM, therapeutic drug monitoring.

^a^ Other induction regimens include agents no longer in routine use, such as OKT3 (muromonab-CD3) and daclizumab (Zenapax), which were administered during the early years of the study period.

Abbreviations: ATG, anti-thymocyte globulin; TDM, therapeutic drug monitoring.

### Univariate and Multivariate Analyses of Risk Factors for the Development of BKPyV-DNAemia Within One Year

Risk factors associated with BKPyV-DNAemia at 1 year were analyzed (Table 2). In the univariate analysis, older age, female sex, body weight, hypertension, pre-dialysis duration, ABO incompatibility, HLA incompatibility, basiliximab induction, ATG induction, tacrolimus TDM, desensitization, and rituximab use had *P* values smaller than 0.1. In the multivariate analysis, older age (OR = 1.02, *P* < 0.001) and longer pre-dialysis duration (OR = 1.02, *P* = 0.023) emerged as significant risk factors for BKPyV-DNAemia positivity at 1 year, while female sex was identified as a protective factor (OR = 0.82, *P* < 0.001). Induction therapy with ATG was significantly associated with an increased risk of BKPyV-DNAemia compared to basiliximab (OR = 3.57, *P* < 0.001). Among CNI regimens, tacrolimus TDM levels of 5–7 ng/mL (OR = 1.64, *P* < 0.001) and ≥7 ng/mL (OR = 1.20, *P* = 0.023) were significantly associated with BKPyV-DNAemia. Additionally, rituximab use showed a marginal association (OR = 1.02, *P* < 0.001).

**TABLE 2:** Univariate and multivariate analyses identifying risk factors for 1-year BK viremia positivity.

	Univariate analysis		Multivariate analysis	
	OR (95% CI)	P-value	OR (95% CI)	P-value
Age, year	1.02 (1.01–1.02)	<0.001	1.02 (1.01–1.02)	<0.001
Female sex	0.86 (0.76–0.98)	0.025	0.82 (0.70–0.95)	0.010
Body weight, kg	1.01 (1.00–1.01)	0.016	1.00 (0.99–1.01)	0.75
Hypertension	1.28 (1.09–1.51)	0.003	1.14 (0.96–1.35)	0.15
Diabetes mellitus	1.12 (0.97–1.29)	0.12	–	–
Pre-transplant Dialysis	1.27 (1.06–1.53)	0.009	1.15 (0.95–1.40)	0.15
Pre-dialysis duration, year	1.03 (1.02–1.04)	<0.001	1.02 (1.00–1.03)	0.026
ABO incompatibility	1.27 (1.07–1.50)	0.005	0.75 (0.44–1.26)	0.27
HLA incompatibility	1.35 (1.05–1.74)	0.019	0.81 (0.48–1.38)	0.44
ATG vs. Basiliximab	4.80 (2.75–8.38)	<0.001	3.57 (2.03–6.27)	<0.001
Cyclosporin	Reference		Reference	–
Tacrolimus TDM <5	0.88 (0.44–1.78)	0.73	0.90 (0.44–1.82)	0.76
5 ≤ Tacrolimus TDM <7	1.46 (1.18–1.81)	<0.001	1.64 (1.31–2.06)	<0.001
Tacrolimus TDM ≥7	1.09 (0.95–1.26)	0.21	1.20 (1.03–1.39)	0.023
Desensitization	1.34 (1.16–1.55)	<0.001	1.68 (1.01–2.77)	0.044
Rituximab	1.38 (1.19–1.60)	<0.001	1.02 (1.01–1.02)	<0.001

Continuous data are presented as means ± standard deviations. Categorical data are presented as a number (%); All continuous variables were analyzed per unit increase: age (per 1 year), body weight (per 1 kg), and pre-dialysis duration (per 1 year).

Abbreviations: ATG, anti-thymocyte globulin; TDM, therapeutic drug monitoring.

### Subgroup Analysis

After excluding 175 patients who lacked sufficient TDM data following BKPyV-DNAemia, a total of 927 patients were categorized into three groups according to the immunosuppressive management: MPA control (n = 579, 62.5%), sirolimus (n = 130, 14.0%), and leflunomide (n = 218, 23.5%). Table 3 presents the baseline characteristics and clinical outcomes among the MPA, sirolimus, and leflunomide groups. The sirolimus group was older (51.4 ± 13.6 years, *P* = 0.021) and had a lower prevalence of ABO incompatibility (13.8%) compared to the other groups (*P* = 0.05). Induction therapy varied significantly across the subgroups (*P* < 0.001), with basiliximab being predominantly used in the MPA and leflunomide groups, while ATG was more common in the sirolimus group. BKPyV-DNA loads at first detection and at peak levels were higher in the leflunomide and sirolimus groups than in the MPA group (*P* < 0.001). CNI withdrawal was observed almost exclusively in the sirolimus group (92.3%, *P* < 0.001). Rejection rates following BKPyV-DNAemia were highest in the sirolimus group (34.4%), followed by leflunomide (22.5%) and MPA (10.9%) (*P* < 0.001). The higher proportion of ATG induction observed in the sirolimus group likely reflects both center-specific induction protocols and the clinical profile of patients selected for sirolimus conversion, who often presented with higher immunologic risk or CNI intolerance.

**TABLE 3:** Characteristics and clinical outcomes of patients treated with MPA, sirolimus, or leflunomide in subgroup analysis.

	MPA	Sirolimus	Leflunomide	*P*-value
Number of patients	579 (62.5)	130 (14.0)	218 (23.5)	
Female sex	222 (38.3)	40 (30.8)	88 (40.4)	0.18
Diabetes mellitus	150 (25.9)	39 (30.0)	59 (27.1)	0.63
Hypertension	483 (83.4)	99 (76.2)	182 (83.5)	0.13
Age, years	49.4 ± 12.0	51.4 ± 13.6	50.1 ± 13.2	0.021
Body weight, kg	61.8 ± 12.7	62.3 ± 12.5	61.1 ± 11.5	0.61
ABO incompatibility	122 (21.1)	18 (13.8)	33 (15.1)	0.05
HLA incompatibility	31 (5.3)	12 (9.2)	14 (6.4)	0.25
Induction therapy				<0.001
None	12 (2.1)	0 (0)	1 (0.5)	
Basiliximab	497 (85.4)	40 (30.8)	183 (84.0)	
ATG	69 (11.9)	89 (68.5)	33 (15.1)	
Other	1 (0.7)	1 (0.8)	1 (0.5)	
First BKV PCR, log copies/mL	3.56 ± 0.9	4.01 ± 1.00	3.69 ± 1.2	<0.001
Maximum BKV PCR, log copies/mL	4.44 ± 1.4	4.98 ± 1.19	5.05 ± 1.34	<0.001
Desensitization	153 (26.4)	29 (22.3)	47 (21.6)	0.29
Rituximab	152 (26.3)	38 (29.2)	46 (21.1)	0.036
Calcineurin inhibitor				
Cyclosporin	61 (10.5)	1 (0.8)	27 (12.4)	<0.001
Tacrolimus TDM^a^ <5	36 (6.2)	3 (2.3)	18 (8.3)	
5 ≤ Tacrolimus TDM^a^ <7	207 (35.8)	4 (3.1)	98 (45.0)	
Tacrolimus TDM^a^ ≥7	275 (47.5)	2 (1.5)	75 (34.4)	
CNI withdrawal	0 (0.0)	120 (92.3)	0 (0.0)	
Treatment failure	78 (13.5)	14 (10.8)	16 (7.3)	0.052
Rejection after BK viremia	63 (10.9)	59 (34.4)	49 (22.5)	<0.001

Continuous data are presented as mean ± standard deviation, while categorical data are presented as number (%).

Abbreviations: MPA; mycophenolic acid; ATG, anti-thymocyte globulin; TDM, therapeutic drug monitoring; CNI, calcineurin inhibitor.

^a^ TDM mean value: from first BKV, positive date to 1 year after.

Among patients included in the subgroup analysis, the median first BKV PCR value was 3.27 log copies/mL (IQR 3.00–4.20), while the median maximum BKV PCR was 4.44 log copies/mL (IQR 3.52–5.40). The median duration of BKPyV DNAemia was 564 days (IQR 259–1,422). These metrics reflect the broad heterogeneity in viral kinetics observed in this population and underscore the need for individualized immunosuppressive strategies.

Notably, among patients with tacrolimus trough levels >5 ng/mL who underwent MPA reduction or discontinuation, no cases were identified in which leflunomide was concurrently initiated. This suggests that leflunomide use in our cohort was generally reserved for patients in whom both MPA and tacrolimus were reduced.

### Risk Factors Associated With BKPyV-DNAemia Treatment Failure and Acute Rejection

Risk factors were analyzed by using univariate and multivariate logistic regression models, including demographic and clinical factors (age, sex, body weight, hypertension, diabetes mellitus, and pre-dialysis duration), immunologic factors (ABO and HLA incompatibility, induction therapy with basiliximab), immunosuppressive management (cyclosporin use, tacrolimus TDM levels <5 ng/mL, 5–7 ng/mL, ≥7 ng/mL, and desensitization with rituximab), BKPyV-DNAemia -related variables (first positive and highest BKPyV-DNA loads), and immunosuppressive regimen groups (MPA [reference], sirolimus, and leflunomide). Variables demonstrating a significance level of *P* <0.1 in univariate analysis were included in the multivariate model.

In the univariate analysis, both the initial and peak BKPyV-DNA loads were independently associated with treatment failure. An OR of 1.33 per log_10_ increase in first viral load indicates a 53% higher risk of persistent viremia for each 10-fold increase in initial BKPyV level. Similarly, an OR of 1.53 for maximum load implies a 33% increased risk per 10-fold rise in peak viral burden. These findings suggest that higher viral replication at presentation and over time both contribute to reduced viral clearance. In the multivariate analysis, BKPyV-DNAemia treatment failure was associated with maximum BKPyV-DNAemia PCR value (OR = 1.56, *P* < 0.001), while CNI withdrawal (OR = 0.05, *P* < 0.001) and the use of leflunomide were associated with a reduced risk (OR = 0.36, *P* = 0.001). Sirolimus use was also significantly associated with a higher risk of treatment failure (OR = 6.25, *P* = 0.007) in multivariate analysis (Table 4).

**TABLE 4:** Univariate and Multivariate analysis of risk factors for BK viremia treatment failure.

	Univariate analysis		Multivariate analysis	
	OR (95% CI)	P-value	OR (95% CI)	P-value
Age, year	1.01 (0.99–1.03)	0.13	–	–
Female sex	0.97 (0.64–1.46)	0.87	–	–
Body weight, kg	1.00 (0.99–1.02)	0.65	–	–
Diabetes mellitus	0.90 (0.57–1.43)	0.66	–	–
Pre-transplant Dialysis	0.63 (0.38–1.06)	0.08	0.63 (0.36–1.11)	0.11
Pre-dialysis duration, year	0.98 (0.93–1.02)	0.30	–	–
ABO incompatibility	0.79 (0.46–1.37)	0.41	–	–
HLA incompatibility	0.89 (0.37–2.12)	0.79	–	–
ATG vs. Basiliximab	0.79 (0.17–3.60)	0.76		
First BKV PCR, log copies/mL	1.33 (1.11–1.59)	0.002	1.07 (0.88–1.31)	0.49
First BKV detection, months	0.95 (0.91–1.06)	0.675	1.01 (0.93–1.10)	0.79
Maximal BKV PCR, log copies/mL	1.53 (1.35–1.75)	<0.001	1.56 (1.36–1.80)	<0.001
Cyclosporin	Reference			
Tacrolimus TDM^a^ <5	0.82 (0.31–2.19)	0.69	0.56 (0.19–1.61)	0.28
5 ≤ Tacrolimus TDM^a^ <7	0.92 (0.47–1.80)	0.81	0.83 (0.41–1.69)	0.61
Tacrolimus TDM^a^ ≥7	0.69 (0.35–1.36)	0.28	0.63 (0.31–1.30)	0.21
CNI withdrawal	0.47 (0.19–1.16)	0.10	0.05 (0.01–0.24)	<0.001
MPA group	Reference			
Sirolimus	0.78 (0.42–1.42)	0.41	6.25 (0.66–23.55)	0.007
Leflunomide	0.51 (0.29–0.89)	0.018	0.36 (0.20–0.66)	0.001
Desensitization	0.85 (0.53–1.37)	0.51	–	–
Rituximab	0.77 (0.47–1.25)	0.29	–	–

Abbreviations: ATG, anti-thymocyte globulin; BKV, BKPyV-DNAemia; CNI, calcineurin inhibitor; TDM, therapeutic drug monitoring; MPA; mycophenolic acid.

^a^ TDM, mean value: from first BKV, positive date to 1 year after.

Table 5 presents the results of univariate and multivariate analyses evaluating the risk factors associated with acute rejection within 1 year following BKPyV-DNAemia. In the multivariate analysis, the maximum BKPyV PCR value (OR = 1.18, *P* = 0.017) was significantly associated with an increased risk of acute rejection, along with tacrolimus TDM <5 ng/mL (OR = 2.65, *P* = 0.033) and CNI withdrawal (OR = 6.00, *P* = 0.044). Leflunomide use was significantly associated with an increased rejection risk (OR = 2.14, *P* < 0.001), while sirolimus use did not show a significant association (*P* = 0.68). An exploratory analysis (Supplementary Figure S1) showed that patients who experienced acute rejection following BKPyV DNAemia had higher initial and peak viral loads compared to those without rejection, suggesting that early and substantial viral replication may contribute to subsequent immunologic injury.

**TABLE 5:** Univariate and multivariate analysis of risk factors for acute rejection within 1 year following BK viremia.

	Univariate analysis		Multivariate analysis	
	OR (95% CI)	P-value	OR (95% CI)	P-value
Age, year	1.02 (1.00–1.03)	0.024	1.01 (0.99–1.03)	0.07
Female sex	0.84 (0.60–1.19)	0.33	–	–
Body weight, kg	1.01 (0.99–1.02)	0.59	–	–
Diabetes mellitus	0.97 (0.67–1.42)	0.89	–	–
Pre-transplant Dialysis	0.88 (0.55–1.40)	0.59	–	–
Pre-dialysis duration, year	1.01 (0.99–1.01)	0.36	–	–
ABO incompatibility	1.00 (0.66–1.54)	0.99	–	–
HLA incompatibility	1.33 (0.70–2.53)	0.38	–	–
ATG vs. Basiliximab	1.01 (0.22–4.63)	0.99		
First BKV PCR, log copies/mL	1.32 (1.13–1.53)	<0.001	1.09 (0.91–1.31)	0.34
Maximal BKV PCR, log copies/mL	1.28 (1.15–1.43)	<0.001	1.18 (1.03–1.36)	0.017
Cyclosporin	Reference			
Tacrolimus TDM^a^ <5	2.53 (1.07–6.01)	0.035	2.65 (1.08–6.51)	0.033
5 ≤ Tacrolimus TDM^a^ <7	1.15 (0.56–2.33)	0.71	1.15 (0.55–2.38)	0.71
Tacrolimus TDM^a^ ≥7	1.04 (0.51–2.10)	0.91	1.23 (0.59–2.55)	0.58
CNI withdrawal	6.42 (3.11–13.26)	<0.001	6.00 (1.05–34.45)	0.044
MPA group	Reference			
Sirolimus	6.81 (4.41–10.50)	<0.001	1.36 (0.27–6.85)	0.68
Leflunomide	2.38 (1.57–3.59)	<0.001	2.14 (1.40–3.29)	<0.001
Desensitization	1.33 (0.92–1.92)	0.13	–	–
Rituximab	1.36 (0.94–1.96)	0.10	1.41 (0.95–2.10)	0.09

Abbreviations: ATG, anti-thymocyte globulin; BKV, BKPyV-DNAemia; CNI, calcineurin inhibitor; TDM, therapeutic drug monitoring; MPA; mycophenolic acid.

^a^ TDM, mean value: from first BKV, positive date to 1 year after.

### Long-Term Graft Survival According to CNI Management

**FIGURE 2:**
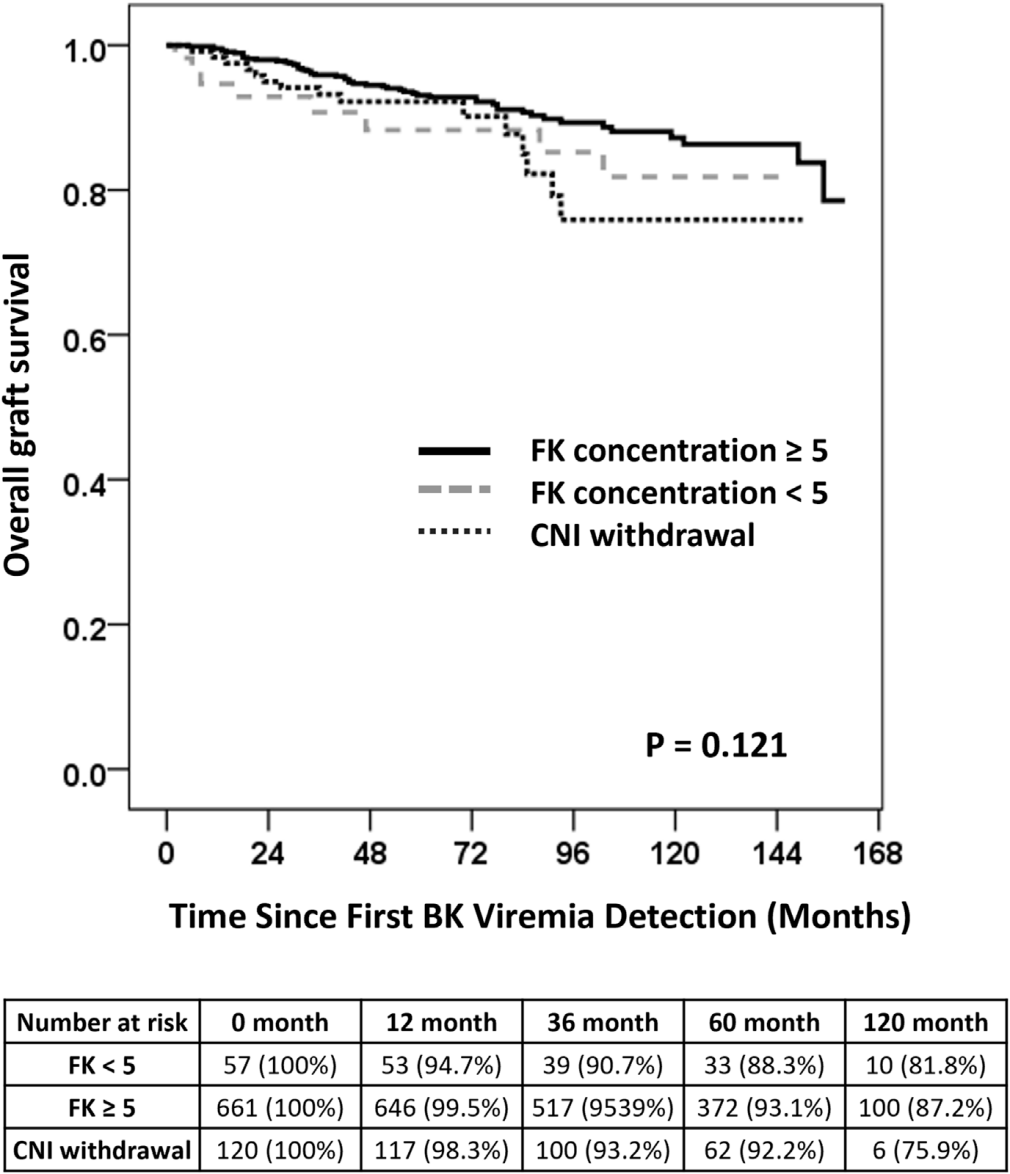
Long-term Graft Survival Following BKPyV-DNAemia. Kaplan-Meier survival curves comparing long-term graft survival among patients stratified by CNI management strategies following BKPyV-DNAemia. The overall log-rank test was not statistically significant (*P* = 0.121). Pairwise comparisons showed that CNI withdrawal was associated with a trend toward worse survival compared to tacrolimus TDM levels of ≥5 ng/mL (*P* = 0.067). Note: No patients with tacrolimus trough levels >5 ng/mL underwent MPA reduction or discontinuation in combination with leflunomide initiation. Leflunomide use was limited to those with simultaneous reduction in both tacrolimus and MPA.

The CNI management groups were categorized as cyclosporin, tacrolimus TDM <5 ng/mL, tacrolimus TDM ≥5 ng/mL, and CNI withdrawal to evaluate long-term graft survival following BKPyV-DNAemia. In the Kaplan-Meier analysis, the overall log-rank test did not show a statistically significant difference in graft survival among the CNI management groups (*P* = 0.121) (Figure 2). Multivariate Cox regression analysis was conducted to identify predictors of graft failure following BKPyV-DNAemia (Table 6). CNI withdrawal was associated with borderline significance for worse survival compared to tacrolimus TDM ≥5 ng/mL (*P* = 0.067). In the multivariate analysis, older age (HR = 1.02, *P* = 0.042), diabetes mellitus (HR = 2.11, *P* = 0.001), and the maximum BKPyV-DNAemia PCR value (HR = 1.24, *P* = 0.001) were identified as significant risk factors for long-term graft failure following BK viremia. Tacrolimus TDM ≥5 ng/mL was associated with a reduced risk of graft failure (HR = 0.54, *P* = 0.036), while CNI withdrawal showed a trend toward a higher risk of graft failure (HR = 4.37, *P* = 0.07). Additionally, sirolimus (HR = 2.12, *P* = 0.003) and leflunomide (HR = 1.94, *P* = 0.006) were associated with a higher risk of graft failure compared to MPA. Patients who experienced graft failure demonstrated higher median first and maximum BKPyV-DNA loads, suggesting that greater early or sustained viral replication may be associated with adverse long-term graft outcomes (Supplementary Figure S2).

**TABLE 6:** Univariate and Multivariate analysis of risk factors for overall graft failure following BK viremia.

	Univariate analysis		Multivariate analysis	
	HR (95% CI)	P-value	HR (95% CI)	P-value
Age, year	1.03 (1.01–1.05)	0.002	1.02 (1.00–1.04)	0.042
Female sex	0.74 (0.48–1.15)	0.18	–	–
Body weight, kg	1.01 (0.99–1.03)	0.32	–	–
Diabetes mellitus	1.83 (1.20–2.81)	0.005	2.11 (1.34–3.34)	0.001
Pre-dialysis duration, year	1.02 (0.98–1.06)	0.48	–	–
ABO incompatibility	0.81 (0.47–1.41)	0.46	–	–
HLA incompatibility	0.73 (0.27–2.00)	0.54	–	–
ATG vs. Basiliximab	0.89 (0.22–3.65)	0.87		
First BKV PCR, log copies/mL	1.15 (0.96–1.37)	0.12	–	–
Maximal BKV PCR, log copies/mL	1.37 (1.22–1.54)	<0.001	1.24 (1.09–1.42)	0.001
Cyclosporin	Reference			
Tacrolimus TDM^a^ <5	0.88 (0.37–2.09)	0.78	0.79 (0.33–1.91)	0.60
5 ≤ Tacrolimus TDM^a^	0.55 (0.31–0.98)	0.044	0.54 (0.30–0.96)	0.036
CNI withdrawal	9.93 (4.75–20.75)	<0.001	4.37 (0.87–22.07)	0.07
MPA group	11.15 (6.31–19.72)	<0.001	1.58 (0.37–6.73)	1.58
Sirolimus	2.62 (1.62–4.24)	<0.001	2.12 (1.29–3.49)	0.003
Leflunomide	2.97 (1.93–4.57)	<0.001	1.94 (1.21–3.11)	0.006
Desensitization	0.96 (0.60–1.55)	0.88	–	–
Rituximab	0.98 (0.61–1.57)	0.93	–	–

Abbreviations: ATG, anti-thymocyte globulin; BKV, BKPyV-DNAemia; CNI, calcineurin inhibitor; TDM, therapeutic drug monitoring; MPA; mycophenolic acid.

^a^ TDM, mean value: from first BKV, positive date to 1 year after.

## Discussion

Our multicenter retrospective study underscores the critical importance of personalized immunosuppressive strategies for managing BKPyV-DNAemia in KT recipients. Key risk factors for BKPyV-DNAemia included older age, induction therapy with ATG, and elevated tacrolimus levels, which should be considered for risk stratification and targeted surveillance. Notably, the balance between preventing rejection and minimizing BKPyV-DNAemia heavily depends on maintaining optimal concentrations of CNI. Maintaining tacrolimus TDM levels at or above 5 ng/mL was associated with a lower risk of graft failure. In contrast, CNI withdrawal, even with the use of sirolimus as an alternative, showed a trend toward increased graft failure. These findings suggest that adequate CNI exposure is crucial for balancing viral control and immune suppression. Substituting MPA with leflunomide effectively reduced BKPyV load but was associated with a higher risk of rejection and inferior long-term graft survival. These findings suggest that prolonged maintenance of leflunomide instead of MPA may increase the risk of acute rejection and compromise graft survival.

Building upon existing literature on tacrolimus TDM [12], we found that tacrolimus trough levels between 5 and <7 ng/mL may represent the “optimal range” to mitigate the risk of BKPyV-DNAemia while maintaining sufficient immunosuppression to prevent rejection. In line with our findings, Schaub et al. demonstrated the effectiveness of a CNI-focused strategy for managing BKV infection in KT recipients by prioritizing tacrolimus reduction. Tacrolimus levels were reduced in a stepwise manner, with adjustments to MMF considered only after achieving sufficient CNI reduction [17]. This approach, supported by evidence of tacrolimus’s inhibitory effect on BKPyV-specific T cells, achieved a 92% clearance rate of BKPyV-DNAemia while maintaining stable allograft function over a median follow-up of 34 months [18, 19]. Moreover, the low clinical rejection rate of 8.6% and stable graft function despite subclinical inflammation further highlight the effectiveness of this strategy. The Kidney Disease Improving Global Outcomes (KDIGO) guidelines recommend reducing MPA first, followed by a reduction in CNI dosage, while the Second International Consensus Guideline presented both antimetabolite-first and CNI-first strategies as viable options. Additionally, KDIGO suggests a general 50% reduction in CNI dosage, whereas the International Consensus Guideline recommends target trough levels of tacrolimus (3–5 ng/mL) and cyclosporine (75–125 ng/mL) [20, 21]. Brennan et al. suggested that reducing antimetabolites before CNI reduction yields similar outcomes in BKPyV-DNAemia clearance compared to direct CNI reduction strategies [22]. These findings indicate that both approaches may be viable, emphasizing the need for individualized adjustments in immunosuppression. Furthermore, based on our study results, minimizing the duration of tacrolimus exposure below 5 ng/mL appears to be the most critical factor in optimizing post- BKPyV-DNAemia outcomes. Notably, large-scale studies stratifying outcomes by CNI levels are lacking, highlighting the significance of our findings in guiding immunosuppressive management.

A recent study from the Swiss Transplant Cohort proposed a five-group classification of BKPyV-DNAemia trajectories based on onset, duration, and clearance patterns [23]. This categorization demonstrated clinical relevance, showing that sustained or recurrent viremia, particularly among early-onset cases, was associated with higher rates of persistent replication and impaired graft function, whereas transient early-onset viremia correlated with more favorable outcomes. To explore this further, we performed a subgroup analysis among patients with early-onset BKPyV-DNAemia (≤90 days post-transplant), stratifying them by whether viremia resolved within 6 months or persisted thereafter. As shown in Supplementary Figure S3, the early-persistent subgroup exhibited a trend toward lower graft survival, although the difference did not reach statistical significance (log-rank p = 0.26). This suggests that duration of viremia may be a more critical determinant of outcome than timing of onset alone. Although we did not formally apply the full trajectory model used in the Swiss study, our findings support the clinical utility of integrating both onset and clearance patterns in future risk stratification frameworks.

Reduction or discontinuation of MPA in the treatment of BKPyV-DNAemia in KT recipients carries a risk of allograft rejection, even after achieving BKPyV-DNAemia clearance. The incidence of acute rejection in patients treated with immunosuppression reduction for BKPyV-DNAemia has been reported to be approximately 10%–30%, with a higher risk observed in patients undergoing more aggressive reductions or conversions to alternative immunosuppressive regimens. [24–26]. In our study, the rejection rates for patients treated with MPA, sirolimus, or leflunomide were 10.9%, 34.4%, and 22.5%, respectively. Notably, the rejection rate in the MPA group (10.9%) was consistent with prior studies that reported rates of 9%–12% for tacrolimus-based regimens combined with MPA or azathioprine [24, 27]. This suggests that reducing immunosuppression in the context of MPA-based regimens can effectively mitigate the risk of rejection while maintaining control of BKPyV-DNAemia The rejection rate in the sirolimus group (34.4%) was significantly higher than that in the MPA group (*P* < 0.001). This discrepancy may be attributed to the elevated initial BKPyV PCR levels in the sirolimus group and the treatment strategy employed at one participating center, where sirolimus was initiated when BKPyV PCR levels reached ≥4, accompanied by the withdrawal of CNI. Consequently, many patients in the sirolimus group underwent CNI withdrawal, which likely contributed to the higher rejection rate. Notably, in the multivariate analysis for acute rejection, sirolimus itself was not identified as a significant risk factor. The leflunomide group exhibited the most effective response to BKPyV-DNAemia treatment, despite having the highest maximum BKPyV-DNAemia PCR levels. However, the risk of acute rejection within 1 year after the onset of BKPyV-DNAemia was 2.1 times higher compared to the MPA group, suggesting that substituting leflunomide may have a similar immunosuppressive effect as withdrawing MPA. These findings indicate that transitioning from MPA to leflunomide can be a highly effective treatment strategy for patients with elevated BKPyV PCR levels. Nevertheless, based on our results, reintroducing a low dose of MPA or maintaining appropriate CNI levels as BKPyV PCR stabilizes may be advisable to minimize the risk of rejection.

Leflunomide is an immunomodulatory and antiviral agent that inhibits dihydroorotate dehydrogenase, thereby suppressing BKPyV replication and lymphocyte proliferation. Its antiviral effects are particularly pronounced in renal tubular epithelial cells, where it reduces the replication of BKPyV by inhibiting DNA synthesis [28, 29]. This dual mechanism allows for a reduction in the use of immunosuppressive drugs without increasing the risk of rejection. While effective in high-risk cases, its use is still associated with rejection and graft dysfunction, and the absence of a clear correlation between serum levels and efficacy complicates its clinical application. Our subgroup analysis suggests that leflunomide is a promising option for managing BKPyV-DNAemia in KT recipients, especially in high-risk cases. Similarly, a study by Aldieri et al. reported a BKPyV-DNAemia clearance rate of 91.4%, including viral eradication in 8 of 11 patients with biopsy-proven BKVN, when leflunomide was used as an adjunct to reduced immunosuppression rather than complete discontinuation of antiproliferative agents [30]. Further evidence from a multicenter study [31] demonstrated a 76% BKPyV-DNAemia clearance rate in KT recipients treated with leflunomide after failing prior therapies. However, 11 patients experienced graft loss, with 9 of these cases attributed to BKVN; rejection episodes occurred in 33% of patients, emphasizing the challenges of balancing immunosuppression and antiviral efficacy. A systematic review [32] corroborated these findings, reporting BKPyV-DNAemia clearance rates ranging from 33% to 92%, although significant heterogeneity in dosing regimens and pharmacokinetics complicated the interpretation of results. Notably, adverse events such as hemolytic anemia and thrombotic microangiopathy were observed, highlighting the importance of monitoring during treatment. Smaller prospective studies further support the efficacy of leflunomide. Faguer et al. reported that 42% of KT recipients with BKVN achieved viral clearance, and 66.6% maintained stable or improved graft function after switching from MMF to leflunomide [33]. Our study suggests a potential role for leflunomide in BKPyV suppression while underscoring the need for careful monitoring to balance efficacy and safety. Study by Bischof et al. summarize contemporary treatment options and emphasize the importance of tailoring immunosuppressive reduction based on viral dynamics histologic severity, and graft function [34]. Their study highlights the limitations of a one-size-fits-all approach and outlines the variable efficacy of adjunctive therapies, such as leflunomide and immunoglobulin, especially in the absence of randomized controlled trials. Our findings align with this perspective, suggesting that while immunosuppressive modulation remains the cornerstone, its optimization requires greater clinical granularity and prospective validation.

The role of sirolimus in managing BKPyV-DNAemia has been highlighted in several studies, demonstrating both antiviral effects and potential benefits in specific patient populations. The TRANSFORM study, a randomized, multicenter trial, evaluated everolimus with reduced exposure to CNIs compared to MPA with standard CNI exposure in *de novo* KT recipients. While not primarily designed to assess BK virus infection, the study reported a significantly lower incidence of BKV replication, based on center-reported data, in the everolimus group compared to the MPA group (8.8% vs. 14.8%, p < 0.001). [35]. Similarly, a retrospective study by Tohme et al. demonstrated a lower incidence of BKPyV-DNAemia in patients converted to sirolimus-based regimens, with clinically significant BKPyV-DNAemia observed in only 4.3% of the sirolimus group compared to 17.9% in the tacrolimus group [36]. These findings suggest a potential role for sirolimus in reducing BKPyV replication, particularly in low-risk populations. The recent BKEver study further supports the effectiveness of early reduction of both MPA and tacrolimus as a first-line approach for managing new-onset BKPyV-DNAemia [37]. In this prospective multicenter cohort, 81.3% of kidney transplant recipients achieved viral clearance within 6 months without an increased incidence of acute rejection. Notably, patients converted to everolimus had a lower clearance rate of 55.7%, suggesting that mTOR inhibitor conversion may be less effective as an initial strategy. These findings are consistent with our results and reinforce the value of a measured, stepwise reduction in immunosuppression for achieving viral control while minimizing rejection risk.

Several *in vitro* studies further support the antiviral properties of sirolimus. One study reported that sirolimus inhibits v replication by impairing mTOR-SP6-kinase activation and suppressing the expression of the BKPyV large T antigen in renal epithelial cells [38]. The inhibitory effects of sirolimus on BKPyV replication were most effective within 24 h of infection, particularly during early viral gene expression, but diminished during the late phase. These findings underscore a potential therapeutic window for sirolimus in the management of BKPyV-DNAemia In contrast, tacrolimus has been shown to activate BK viral replication via the same FKBP-12 pathway, highlighting a mechanistic divergence that could inform tailored immunosuppressive strategies. Moreover, sirolimus may modulate the immune response to the BKPyV through its effects on T-cell function. A study by Araki et al. demonstrated that rapamycin (sirolimus) enhances the formation of memory CD8^+^ T cells, which exhibit superior antiviral functionality, higher expression of markers associated with long-lived immunity (e.g., CD127, CD62L, Bcl-2), and reduced expression of senescence markers such as KLRG-1 [39]. These findings suggest that sirolimus may augment the antiviral immune response while providing essential immunosuppression for transplant recipients. However, complete withdrawal of CNIs when using sirolimus, particularly in immunologically high-risk patients, may increase the risk of acute rejection and negatively impact long-term graft survival.

Our study also highlights the wide variability in BKPyV DNAemia dynamics. The median duration of DNAemia exceeded 1.5 years in our cohort, with some patients experiencing persistence for more than 4 years. The initial and peak viral loads were notably higher in patients requiring alternative immunosuppressive regimens such as sirolimus or leflunomide. These findings reinforce the notion that viral kinetics—not just presence or absence of viremia—may influence both treatment decisions and graft outcomes, and should therefore be considered in future prospective stratification models.

Interestingly, our study found that female sex was associated with a lower risk of developing BK viremia, which complements prior observations identifying male sex as a potential risk factor, such as those noted in the The Transplantation Society (TTS) guidelines [21]. Although the underlying mechanisms remain unclear, pharmacokinetic studies have reported that female recipients tend to exhibit higher tacrolimus exposure and slower clearance, which may affect immunosuppressive intensity and susceptibility to viral reactivation [40, 41]. Additionally, sex-based differences in antiviral immunity have been described. These biological factors may contribute to the observed association, though further investigation is warranted to clarify causality.

Prolonged pre-transplant dialysis duration may, in part, reflect underlying immunologic barriers—such as HLA or ABO incompatibility—that delay transplantation and potentially influence post-transplant infection risk. However, in our cohort, there was no statistically significant association between pre-transplant dialysis and the need for desensitization (Pearson χ^2^ = 1.684, P = 0.194), suggesting that pre-dialysis status was not primarily driven by immunologic risk factors.

The newly published consensus standard for BKPyV-associated nephropathy recommends not only timely reduction of immunosuppression upon BKPyV-DNAemia detection but also careful re-escalation of maintenance immunosuppression once viral clearance is achieved [21, 42]. In our retrospective cohort, data on post-clearance immunosuppressive intensification—including MPA reintroduction or increased tacrolimus dosing—were not consistently recorded. Moreover, substitution of MPA with leflunomide—a less potent immunosuppressant—may leave patients functionally under-immunosuppressed, potentially contributing to late acute rejection and graft loss. This observation highlights the need for closure of the immunosuppressive gap following viral clearance, ideally in line with expert guideline recommendations.

Despite its large sample size and extended follow-up, this study has several limitations. Its retrospective design and focus on Korean transplant centers may limit the generalizability of the findings to other populations. Normalization of BKV PCR values, such as using fold-change relative to the assay’s lower limit of detection, can enhance cross-center comparability in multicenter studies. In our cohort, however, the distribution of viral load values was empirically consistent across institutions, supporting the validity of using absolute values for analysis without additional transformation. Additionally, transplant practices evolved over the 15-year study period, potentially introducing unmeasured confounders. Variability in BKPyV detection and management protocols across centers may have resulted in selection bias, particularly in the sirolimus group, where one center exclusively implemented MPA discontinuation, sirolimus initiation, and complete CNI withdrawal for patients with BKPyV PCR levels greater than 4. This approach likely resulted in more severe or refractory BKPyV infections at baseline, influencing treatment outcomes despite multivariate adjustments. We therefore attempted to address these biases through comprehensive multivariate analyses to ensure robust findings. Moreover, our cohort included a relatively high proportion of immunologically high-risk patients, which may have impacted both BKPyV-DNAemia incidence and rejection patterns compared to lower-risk populations. These factors highlight the complexity of immunosuppressive modifications in BKPyV-DNAemia management and underscore the need for individualized treatment strategies based on patient-specific risk profiles. We acknowledge that immune reconstitution after immunosuppression reduction may lead to antiviral inflammatory infiltrates that mimic T-cell mediated rejection (TCMR). In our study, rejection diagnoses were based on local Banff assessments without centralized or molecular review, limiting our ability to distinguish true TCMR from beneficial antiviral responses. This represents a limitation and highlights the need for more refined biopsy evaluation in future studies. Lastly, corticosteroid exposure, including pulse therapy for acute rejection, was not uniformly documented across centers and could not be systematically analyzed. While most centers followed standard protocols, the lack of detailed data on cumulative steroid burden is a limitation that future studies should address.

Another limitation of our study is the composition of the sirolimus group. The vast majority (92.3%) of patients receiving sirolimus were managed in a CNI-withdrawal setting, with only 10 patients receiving sirolimus in combination with tacrolimus. No patients received cyclosporine plus sirolimus. Although the sirolimus + TAC subgroup showed a significantly higher rate of BKPyV treatment failure (40.0% vs. 8.3%, p = 0.002) but a lower rate of 1-year rejection (20.0% vs. 47.5%, p = 0.086) compared to the CNI-free sirolimus group, as shown in Supplementary Table S1, these results should be interpreted with caution due to the small sample size and the relatively high tacrolimus trough levels (mean TDM 7.6 ng/mL). Therefore, our study is not adequately powered to assess the effects of standard low-dose CNI + mTORi regimens and may not reflect their clinical efficacy.

This multicenter retrospective study highlights key risk factors for BKV and offers guidance on immunosuppressive strategies in kidney transplant recipients. Maintaining tacrolimus trough levels between 5 and 7 ng/mL balances BKPyV-DNAemia control and rejection risk. Adjusting or replacing MPA, including with leflunomide, may aid BKPyV-DNAemia management but carries long-term immunologic considerations. While CNI withdrawal may promote viral clearance, it raises rejection risk. We recommend individualized adjustment of immunosuppression based on BKPyV PCR trends. These findings support more personalized management approaches to improve long-term outcomes. Future prospective studies and incorporation of molecular diagnostics may enhance risk prediction and treatment optimization.

## Data Availability

The raw data supporting the conclusions of this article will be made available by the authors, without undue reservation.
