# Supplementary material for: Risk Prediction and Management of BKPyV-DNAemia in Kidney Transplant Recipients: A Multicenter Analysis of Immunosuppressive Strategies
Source: Transpl Int. 2025 Jul 17;38:14738. doi: 10.3389/ti.2025.14738 (PMC12327399; doi:10.3389/ti.2025.14738)
Supplement: Supplementary file 1 [file DataSheet1.docx]

**Supplementary Figure 1.** Comparison of first and maximum BKPyV-DNA loads (log₁₀ copies/mL) between patients with and without acute rejection. Patients who experienced rejection exhibited higher median viral loads at both time points, supporting a potential link between early viral burden and rejection risk.

**
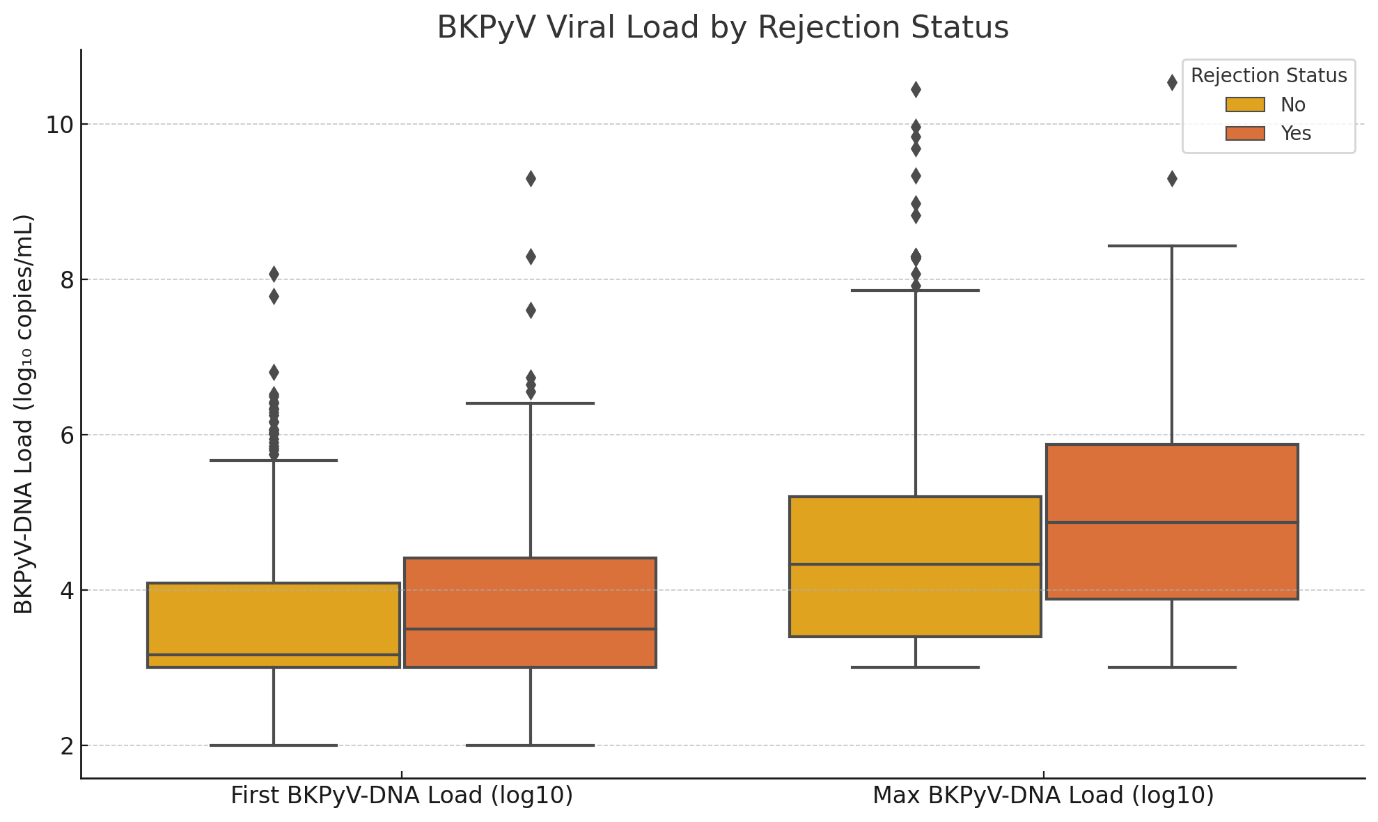
**


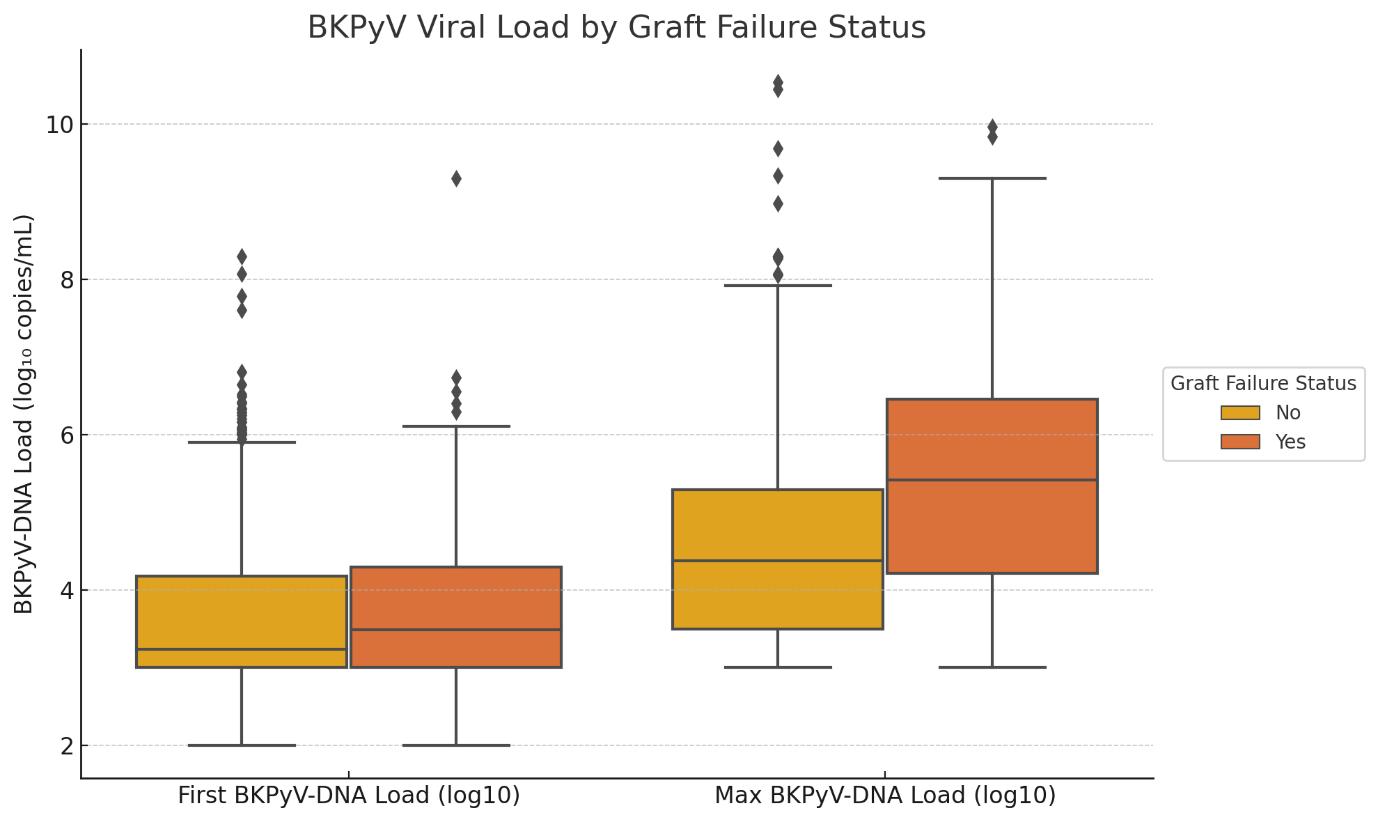
**Supplementary Figure 2.** Comparison of first and maximum BKPyV-DNA loads (log₁₀ copies/mL) between patients with and without graft failure. Patients who experienced graft failure demonstrated higher median values for both initial and peak BKPyV-DNA loads compared to those with preserved graft function, suggesting that increased viral burden may be associated with long-term allograft injury.
